# Enhanced Stability of Complex Sound Representations Relative to Simple Sounds in the Auditory Cortex

**DOI:** 10.1523/ENEURO.0031-22.2022

**Published:** 2022-08-01

**Authors:** Harini Suri, Gideon Rothschild

**Affiliations:** 1 Department of Psychology, University of Michigan, Ann Arbor, Michigan 48109; 2 Kresge Hearing Research Institute, Department of Otolaryngology-Head and Neck Surgery, University of Michigan, Ann Arbor, Michigan 48109

**Keywords:** auditory cortex, plasticity, stability

## Abstract

Typical everyday sounds, such as those of speech or running water, are spectrotemporally complex. The ability to recognize complex sounds (CxSs) and their associated meaning is presumed to rely on their stable neural representations across time. The auditory cortex is critical for the processing of CxSs, yet little is known of the degree of stability of auditory cortical representations of CxSs across days. Previous studies have shown that the auditory cortex represents CxS identity with a substantial degree of invariance to basic sound attributes such as frequency. We therefore hypothesized that auditory cortical representations of CxSs are more stable across days than those of sounds that lack spectrotemporal structure such as pure tones (PTs). To test this hypothesis, we recorded responses of identified layer 2/3 auditory cortical excitatory neurons to both PTs and CxSs across days using two-photon calcium imaging in awake mice. Auditory cortical neurons showed significant daily changes of responses to both types of sounds, yet responses to CxSs exhibited significantly lower rates of daily change than those of PTs. Furthermore, daily changes in response profiles to PTs tended to be more stimulus-specific, reflecting changes in sound selectivity, compared with changes of CxS responses. Last, the enhanced stability of responses to CxSs was evident across longer time intervals as well. Together, these results suggest that spectrotemporally CxSs are more stably represented in the auditory cortex across time than PTs. These findings support a role of the auditory cortex in representing CxS identity across time.

## Significance Statement

The ability to recognize everyday complex sounds such as those of speech or running water is presumed to rely on their stable neural representations. Yet, little is known of the degree of stability of single-neuron sound responses across days. As the auditory cortex is critical for complex sound perception, we hypothesized that the auditory cortical representations of complex sounds are relatively stable across days. To test this, we recorded sound responses of identified auditory cortical neurons across days in awake mice. We found that auditory cortical responses to complex sounds are significantly more stable across days compared with those of simple pure tones. These findings support a role of the auditory cortex in representing complex sound identity across time.

## Introduction

Everyday sounds such as human speech, animal vocalizations, the sound of running water or rustling of leaves, are spectrotemporally complex (Ehret and Haack, 1982; Doupe and Kuhl, 1998; Gygi et al., 2007). A key brain region involved in the perception of spectrotemporally complex sounds is the auditory cortex (AC; Rauschecker, 1998; Griffiths et al., 2004; Nelken, 2004, 2008; Nelken and Bar-Yosef, 2008; Bizley et al., 2009; King et al., 2018; Maor et al., 2020). For example, AC lesions result in a more profound impairment in processing complex sounds (CxSs) in comparison with pure tones (PTs) and other simple sounds in both humans (Kaga et al., 1997; Griffiths, 2012) and animal models (Ohl et al., 1999; Harrington et al., 2001; Rybalko et al., 2006). Responses of AC neurons to CxSs can often not be predicted from a linear combination of responses to the PT components of the CxS (Nelken et al., 1999; Barbour and Wang, 2003; Wang et al., 2005; Atencio et al., 2008; Sadagopan and Wang, 2009; Schreiner et al., 2011; Mizrahi et al., 2014; Harper et al., 2016; Angeloni and Geffen, 2018; Schwartz et al., 2020). Furthermore, studies using a range of approaches have shown that AC responses to CxSs represent sound “identity” with a substantial invariance to its frequency components and other acoustic parameters (Chechik and Nelken, 2012; Nelken et al., 2003, 2014; Carruthers et al., 2015; Blackwell et al., 2016; Town et al., 2018; Harpaz et al., 2021). While these studies suggest an important role of the AC in representing the identity and meaning of CxSs, to what degree these representations are stable across time remains unknown.

To support the ability to recognize sensory stimuli and their associated meaning, the neural representations of the stimuli are expected to be stable across time (Lütcke et al., 2013; Schoonover et al., 2021). At the large-scale spatial resolution, the representation of tone frequency across the AC tonotopic map is indeed generally stable in adulthood in the absence of instructive learning or manipulation of the acoustic environment (Merzenich et al., 1976; Guo et al., 2012). At the single-cell level, receptive fields of most auditory cortical neurons have been found to be stable across up to 2 h of recording, though a minority of neurons exhibited significant changes within this time frame (Elhilali et al., 2007). However, whether AC sound representations are stable across days and whether the representations of CxSs and PTs are similarly stable, remains unknown. Given the suggested involvement of AC in representing CxS identity, we hypothesized that CxSs would be more stably represented in the AC across time compared with PTs. Here, we tested this hypothesis by recording the responses of identified layer 2/3 (L2/3) AC excitatory neurons to both PTs and CxSs across days in awake mice using two-photon calcium imaging.

## Materials and Methods

All animal procedures were performed in accordance with the regulations of the University of Michigan animal care committee.

### Animals

We used 13 Thy1-GCaMP6f mice [C57BL/6J-Tg (Thy1-GCaMP6f) GP5.17Dkim/J; catalog #025393, The Jackson Laboratory; 10 males, 3 females; age, 8–15 weeks], which express the GCaMP6f calcium indicator in excitatory pyramidal neurons (Dana et al., 2014). Mice were housed under a reverse 12 h light/dark cycle, with lights on at 8:30 P.M. Experiments were conducted between 11:00 A.M. and 4:00 P.M., and each animal was imaged around the same time of day across all days of data collection so that the time gap between consecutive imaging days was ∼24 h.

### Surgical procedure

All surgeries were performed on mice anesthetized using ketamine (100 mg/kg, i.p.) and xylazine (10 mg/kg, i.p.). Anesthetized mice were placed in a stereotaxic frame (catalog #514, Kopf Instruments), and injections of an anti-inflammatory drug (carprofen, 5 mg/kg, s.c.) and a local anesthetic (lidocaine, s.c.) were administered. A craniotomy was performed over the right primary AC (anteroposterior, −3.1 mm; mediolateral, 4.6 mm; lateral from midline; Extended Data Fig. 1-1) using a 3 mm biopsy punch (Integra), and a 3-mm-diameter round glass cranial window was secured over this craniotomy. A custom-made lightweight (<1 g) titanium head bar was attached to the left side of the skull using dental cement and cyanoacrylate glue to allow for head-fixed imaging. During the surgery, body temperature was maintained at 38°C, and the depth of anesthesia was regularly assessed by checking the pinch withdrawal reflex. Mice were treated with carprofen for 48 h postsurgically and allowed to recover for a week.

### Two-photon calcium imaging

Mice were first habituated to the imaging setup and the sound protocols for 3 d. During the 3 d habituation period, the animals were exposed to the same PT and CxS stimuli as during imaging days 1–5 while being head-fixed in the same setup under the two-photon microscope while being positioned on a circular treadmill (without imaging). Each stimulus was presented 30–35 times in total across the 3 d habituation period.

During imaging, the objective of the microscope was placed perpendicular to the surface of the cranial window to access the AC. Imaging was conducted using a two-photon microscope (model Ultima IV, Bruker) through water-immersion objectives [40×: numerical aperture (NA) = 0.65 (*n* = 2 mice); 16×: NA = 0.8 (*n* = 11 mice); Nikon], and a pulsed laser was used to provide excitation at 940 nm (MaiTai eHP DeepSee, Spectra Physics). Data were collected using galvanometric (“galvo”) scanning of 256 × 256 pixel images at 3 frames/s. We conducted a separate set of recordings from the same neurons using galvo scanning and faster resonant scanning at 60 frames/s (averaging every 4 frames to yield 15 frames/s) and found that responsiveness, response magnitude, and trial-to-trial consistency were not underestimated by the slower galvo imaging sample rate (Extended Data Fig. 1-2). During the period of habituation, focal planes with a high yield of neurons were determined in L2/3 (imaged at depths of 150–330 μm; Meng et al., 2017). The overlying blood vessel patterns and position with respect to the cortical surface were noted and were used to image the same focal planes across 5 consecutive days of the experiment.

### Auditory stimuli

**Figure 1.**
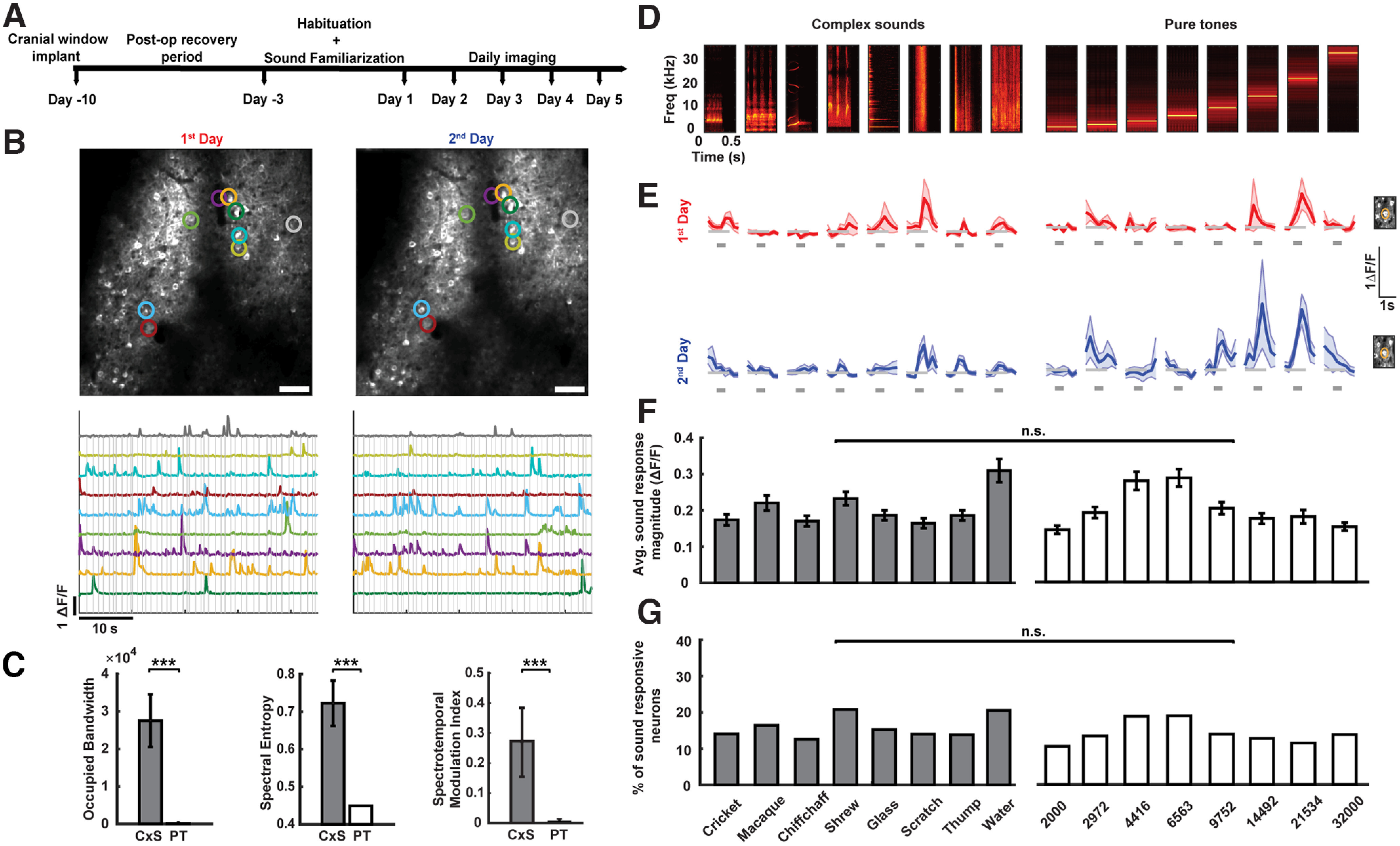
Imaging responses of identified L2/3 auditory cortical excitatory neurons to pure tones and complex sounds across days. ***A***, An illustration of the experimental timeline. ***B***, Top, Example two-photon micrographs from L2/3 of the auditory cortex in an awake mouse from 2 consecutive days. Scale bar, 60 μm. Colored circles identify example individual neurons matched across 2 consecutive days. Extended Data Figure 1-1 shows example histological verification of the imaging location. See Extended Data Figure 1-4 for validation of neuron matching across days. Bottom, Δ*F*/*F* traces of the neurons marked in the micrographs during a pure tone protocol. Gray lines indicate the stimuli. Calibration: 10 s, 1 Δ*F*/*F*. ***C***, Comparison of spectrotemporal features of the CxS and PT. From left to right: occupied bandwidth (***p = 0.00015, Mann–Whitney *U* test), spectral entropy (***p = 0.00015 Mann–Whitney *U* test), and spectrotemporal modulation index (***p = 0.00015, Mann–Whitney *U* test). ***D***, Spectrograms of the sound stimuli presented in the complex sounds (left) and pure tone (right) protocols. The identity of the complex sounds and the frequency of the pure tones are labeled at the bottom of ***G***. ***E***, Responses of a representative neuron to complex sounds and pure tones (the stimuli correspond to ***D*** column-wise) across 2 consecutive days (first day in red, second day in blue). Shaded area marks the mean ± SEM across trials. The gray bar below each response indicates the stimulus time (0.5 s). Calibration: 1 s, 1 Δ*F*/*F*. The cell body of the neuron as imaged during the 2 d is highlighted on the right. Extended Data Figure 1-2 validates that responses included in the study were not underestimated because of sampling rate, and Extended Data Figure 1-3 shows the distribution of the number of trials included in the data. ***F***, Average sound response magnitude (mean Δ*F*/*F* across all trials over the stimulus window) across all days of imaging in response to complex sounds and pure tones (the stimuli correspond to ***D***–***G*** column-wise). Error bars indicate the mean ± SEM. Number of neurons across all pairs of consecutive days (with repetitions): CxSs, 557; PTs, 587; *p* = 0.98 (two-way ANOVA). ***G***, Percentage of sound-responsive neurons recorded across all days of imaging in response to complex sounds and pure tones (the stimuli correspond to ***D***–***G*** column wise). Number of neurons across all pairs of consecutive days (with repetitions): CxSs, 557; PTs, 587, *p* = 0.875 (bootstrap test, see Materials and Methods; Table 1). Sound stimuli for each are indicated below: eight complex sounds and eight pure-tone frequencies (in hertz).

Stimuli were generated at a sampling rate of 97.6 kHz using MATLAB and presented to the animal using an SA1 speaker amplifier, an ED1 speaker driver, and a multifield magnetic speaker (MF1) positioned ∼10 cm in front of the animal, all by Tucker-Davis Technologies. Acoustic stimuli consisted of the following two protocols: PTs consisted of eight pure tone stimuli at 2–32 kHz (Fig. 1*D*), while CxSs consisted of four animal vocalizations (cricket, macaque, chiffchaff, and water shrew) and four environmental sounds (glass, thump, scratch, and water; Fig. 1*D*). The CxSs had significantly higher-frequency bandwidth, spectral entropy, and spectrotemporal modulation compared with the PTs (Fig. 1*C*). The duration of each sound was 500 ms (padded with silence for some of the CxSs) and sound intensity was 65–70 dB SPL. In a given imaging session for each focal plane, each sound within a protocol was repeated 10 times in a pseudorandom order with an interstimulus interval of 1.5 ± 0.3 s. The order of the sound protocols was shuffled across experiments.

10.1523/ENEURO.0031-22.2022.f1-1 Figure 1-1 Histological verification of imaging location. Representative images of coronal brain sections from three animals used in this study. Following the completion of the experiments, mice were killed with an overdose of xylazine (10 mg/kg, i.p.) and the imaging cranial window was removed. Using a nanoFil needle (Hamilton), we injected 1 μl of Dil Tracer (catalog #D282, Thermo Fisher Scientific) into the site of imaging identified by blood vessel patterns and covered the brain surface for 5 min to maximize labeling and prevent fluorescence loss caused by the perfusion during tissue fixation with 4% PFA. The extracted brains were kept in PFA for 3 d and then transferred to 30% sucrose solution for another 3–4 d before cryosectioning. The brains were sliced in 50-μm-thick sections and preserved with Fluoroshield mounting medium with DAPI (Abcam). Recording site confirmation was done by imaging tissue sections positive for GCaMP, DAPI, and Dil fluorescence. Dil fluorescence trace from brain sections were cross-referenced with the Allen Mouse Common Coordinate Framework using NeuroInfo software (MBF Bioscience). The location of the cranial window is indicated on each brain slice, and the specific site of imaging is marked by DiI in yellow. Scale bar, 1000 μm. Download Figure 1-1, TIF file.

**Figure 2.**
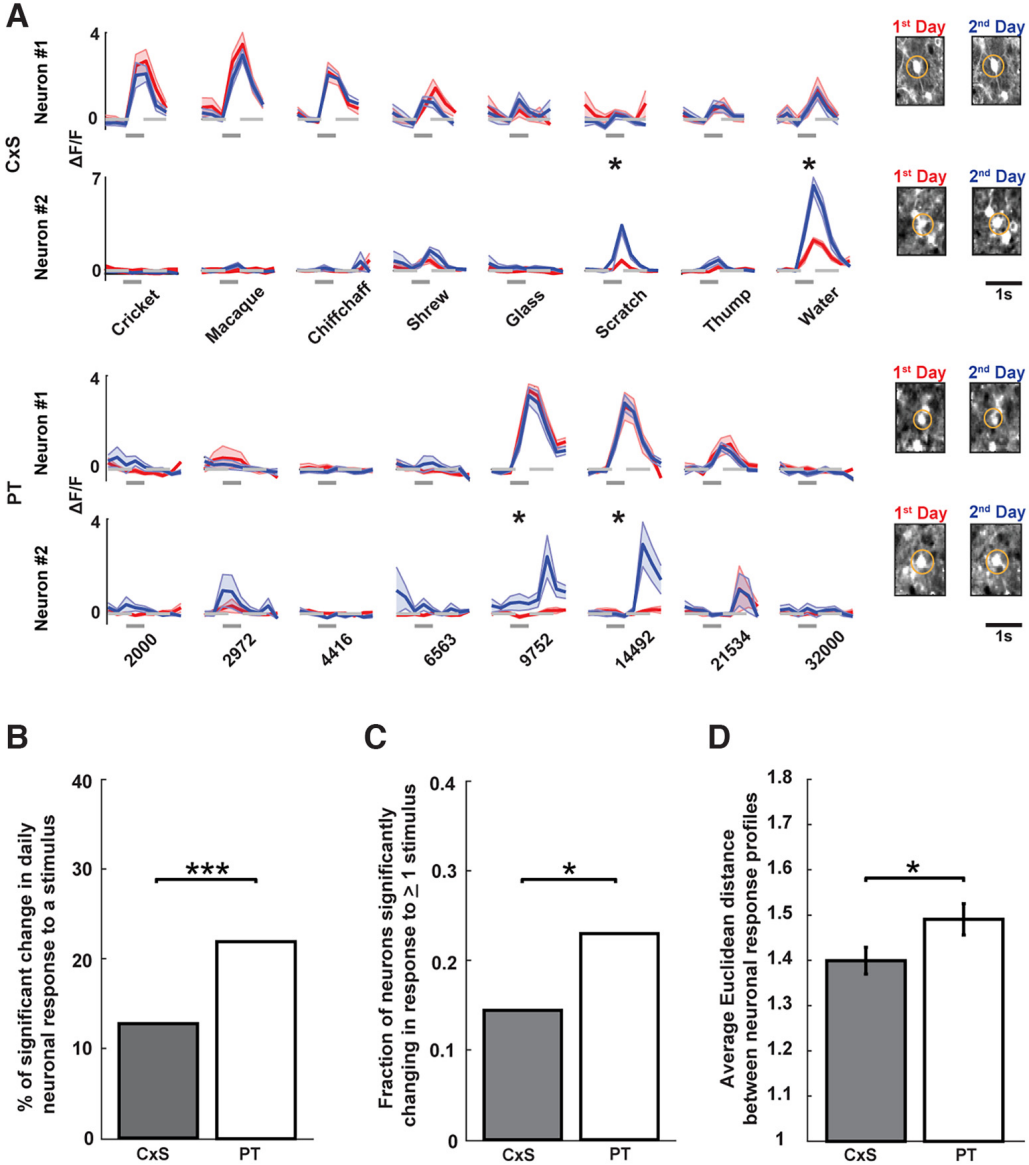
Auditory cortical responses to complex sounds are more stable than those to pure tones across days. ***A***, Responses of two representative neurons to complex sounds (rows 1 and 2) and pure tones (rows 3 and 4) across 2 consecutive days (first day in red, second day in blue). Shaded area marks the mean ± SEM across trials. Gray bars indicate the stimulus time. Stars indicate significant response changes (see Materials and Methods). Calibration: 1 s. The cell bodies of the imaged neurons are shown on the right. Rows 1 and 3 show neuronal responses that were stable from one day to the next, and rows 2 and 4 show neuronal responses that changed from one day to the next. ***B***, Percentage of significant changes in response to complex sounds and pure tones across pairs of consecutive days. CxSs: 12.15% (66 of 543); PTs: 22.01% (114 of 518); ***p = 1.91 × 10^−5^ (χ^2^ test). ***C***, Fraction of neurons that show a significant change in response to at least one stimulus across pairs of consecutive days. CxSs: 0.14 (34 of 241); PTs: 0.23 (59 of 256); *p = 0.011 (χ^2^ test). ***D***, Average Euclidean distance between the response profile of a neuron (to either CxSs or PTs) from one day to the next day; *p = 0.046 (two-sided *t* test). Extended Data Figure 2-1 shows the relationship between the changes in responsiveness of a neuron to CxS and PT.

10.1523/ENEURO.0031-22.2022.f1-2 Figure 1-2 Comparison of auditory cortical responses to CxS and PT in galvo and resonant scanning modes. ***A***, Responses of three representative neurons in galvo (in maroon) and in resonant (in green) scanning modes to CxS and PT stimuli (corresponding stimuli are indicated at the bottom of the panel). Shaded area marks the mean ± SEM across trials. The gray bar below each response indicates the stimulus time (0.5 s). Calibration: 1 s. The cell body of the neuron as imaged in the two scanning modes is highlighted on the right. ***B***, Average sound response magnitude (mean Δ*F*/*F* across all trials over the stimulus window) across both imaging modes in response to complex sounds (Stimuli #1–8) and pure tones (Stimuli #9–16). Error bars indicate the mean ± SEM. Each focal plane was imaged in galvo and resonant scanning modes alternating twice, and all pairs of consecutive imaging sessions were included in the comparison between galvo and resonant scanning modes. The number of neurons across all pairs of scanning modes (with repetitions): galvo, 576; resonant, 540; *p* = 0.044 (two-way ANOVA). In a separate analysis, we found that the likelihood of neurons to be responsive to a specific CxS and a specific PT using resonant scanning was 7.74% (41 of 530) and 7.24% (38 of 525), respectively. Using galvo scanning, these values were slightly higher, at 9.23% (53 of 574) and 10.44% (55 of 527), respectively, suggesting that significant responses were not underestimated by the use of galvo scanning. ***C***, Correlation of the response magnitudes of individual responsive neurons (mean Δ*F*/*F* across all trials over the stimulus window) to individual stimuli (both CxS and PT included) in galvo and resonant scanning modes. Neurons were matched across scanning modes using an automated MATLAB algorithm (https://github.com/ransona/ROIMatchPub) and then validated by visual inspection. Dashed gray line represents the diagonal. Correlation coefficient = 0.5, *p* = 2.36 × 10^−14^ (Pearson’s correlation). ***D***, Distribution of the difference in response magnitude between galvo and resonant scanning modes across neurons and stimuli (corresponding to the difference between the X and Y values of the points in ***C***; *p* = 1.6 × 10^−6^, Wilcoxon signed-rank test). ***E***, Distributions of the trial-by-trial variance in response magnitude to given stimuli in galvo (maroon) and resonant (green) scanning modes. *p* = 0.09 (Mann–Whitney *U* test). Download Figure 1-2, TIF file.

**Table 1:** Summary of statistical analysis used in this study

Figure	Data structure	Type of test	Statistical data
1*C*	Non-normal	Mann–Whitney *U* test	First panel: rank sum = 36; *p* = 0.00015 Second panel: rank sum = 36; *p* = 0.00015 Third panel: rank sum = 100; *p* = 0.00015
1*E*	Normal distribution with two factors	Two-way ANOVA	F1: *F* = 0; df = 1; *p* = 0.98 F2: *F* = 6.86; df = 7; *p* < 0.0001
1*F*	Non-normal	Bootstrap test (describedabove)	10,000 randomly simulated proportions for one group (PT), given the probability of the other group (CxS). *p* = 0.875
2*B,C*	Non-normal	χ^2^ test for proportions and confirmed by Fisher’s exact test	Figure 2*B*: CXS = 12.15% (66 of 543); PTs = 22.01% (114 of 518); χ^2^ statistic = 18.27; *p* = 1.91e^−5^, Fisher’s exact test: odds ratio = 49.03%; CI = 35.22%, 68.26% Figure 2*C*: CXS = 0.14 (34 of 241); PTs = 0.23 (59 of 256); χ^2^ statistic = 6.52; *p* = 0.011 Fisher’s exact test: odds ratio = 0.55; CI = 0.3445, 0.8730
2*D*	Normal distribution	Two-sided *t* test	*t* = −2.0023; *p* = 0.046; df = 495; SD = 0.51; CI = −0.1809, −0.0017
3*B*	Non-normal	*z* test for proportions	*z* value = −1.7811; *p* = 0.037
3*C*	Non-normal	Mann–Whitney *U* test	*z* value = −2.8857; *p* = 0.0039
4*A,B*	Non-normal	Bootstrap test (described above)	10,000 randomly simulated proportions for one group (PT), given the probability of the other group (CxS) computed for each sub category Figure 4*A*: *p* = 0.0015 Figure 4*B*: *p* = 0.1084
4*C*	Normal distribution with two factors	Two-way ANOVA with interaction	F1: *F* = 9.52; df = 3; *p* < 0.001 F2: *F* = 5.89; df =1; *p* = 0.015 F1 * F2: *F* = 0.1; df = 3; *p* = 0.961

**Figure 3.**
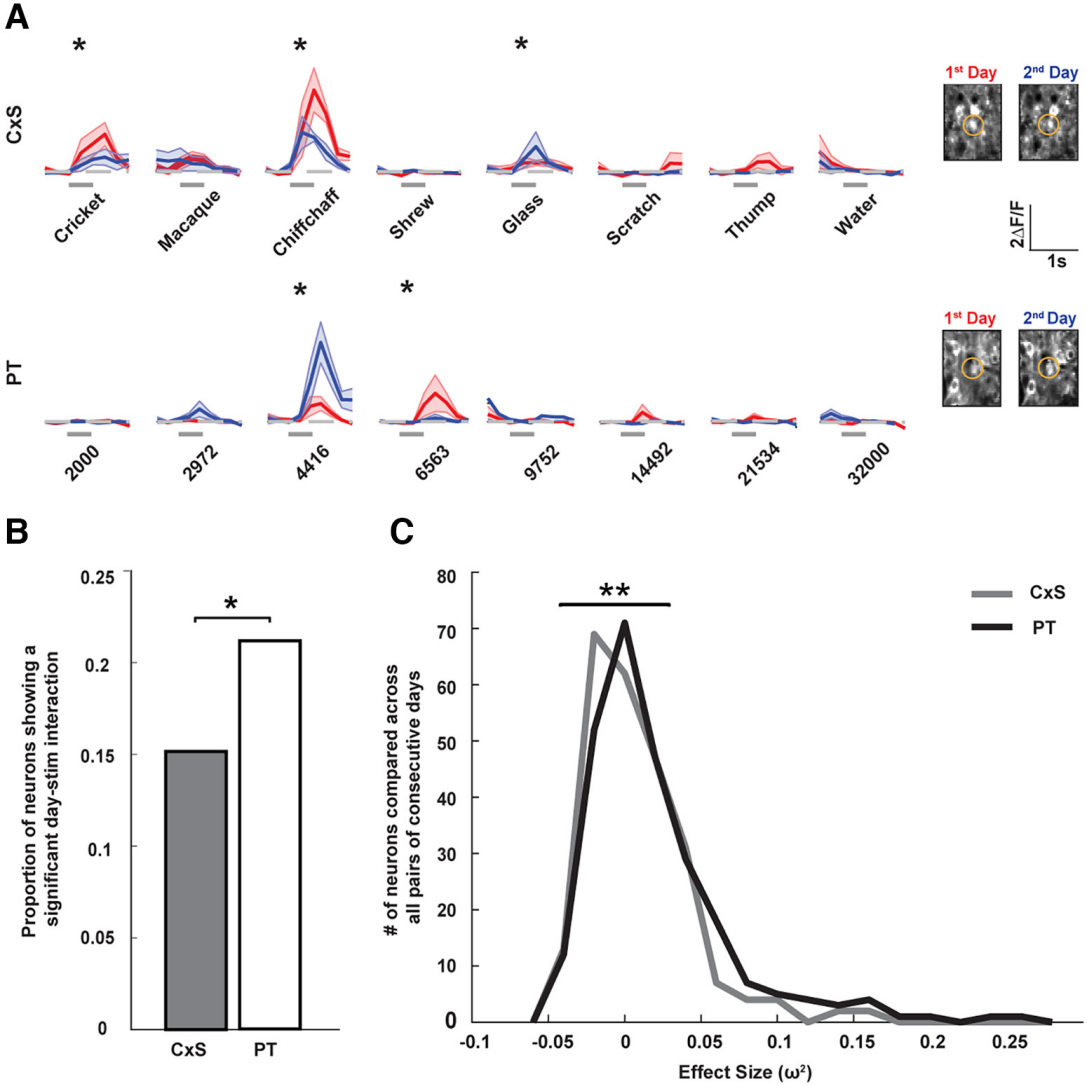
Daily plasticity in responses to pure tones is more stimulus-specific than to complex sounds. ***A***, Responses of two representative neurons to CxSs (row 1) and PTs (row 2) across 2 consecutive days, showing stimulus-specific changes. Shaded area marks the mean ± SEM across trials. Gray bars indicate stimulus timing. Stars indicate significant response changes. Calibration: 1 s, 2 Δ*F*/*F*. The cell bodies of the imaged neurons are shown on the right. ***B***. Proportion of neurons across all pairs of consecutive days showing a significant day–stimulus interaction, computed via two-way ANOVA. CxSs: 14.9% of neurons (36 of 241); PTs: 21.1% of neurons (54 of 256); *p = 0.037 (*z* test for proportions). ***C***, Distributions of the effect size (ω^2^) indicating the strength of the interaction between day and stimulus for CxSs (gray) and PTs (black) for each neuron across all consecutive days. **p = 0.0039 (Mann–Whitney *U* test).

10.1523/ENEURO.0031-22.2022.f1-3 Figure 1-3 Distribution of number of trials included per stimulus across the dataset following trial exclusion due to locomotion. Download Figure 1-3, TIF file.

**Figure 4.**
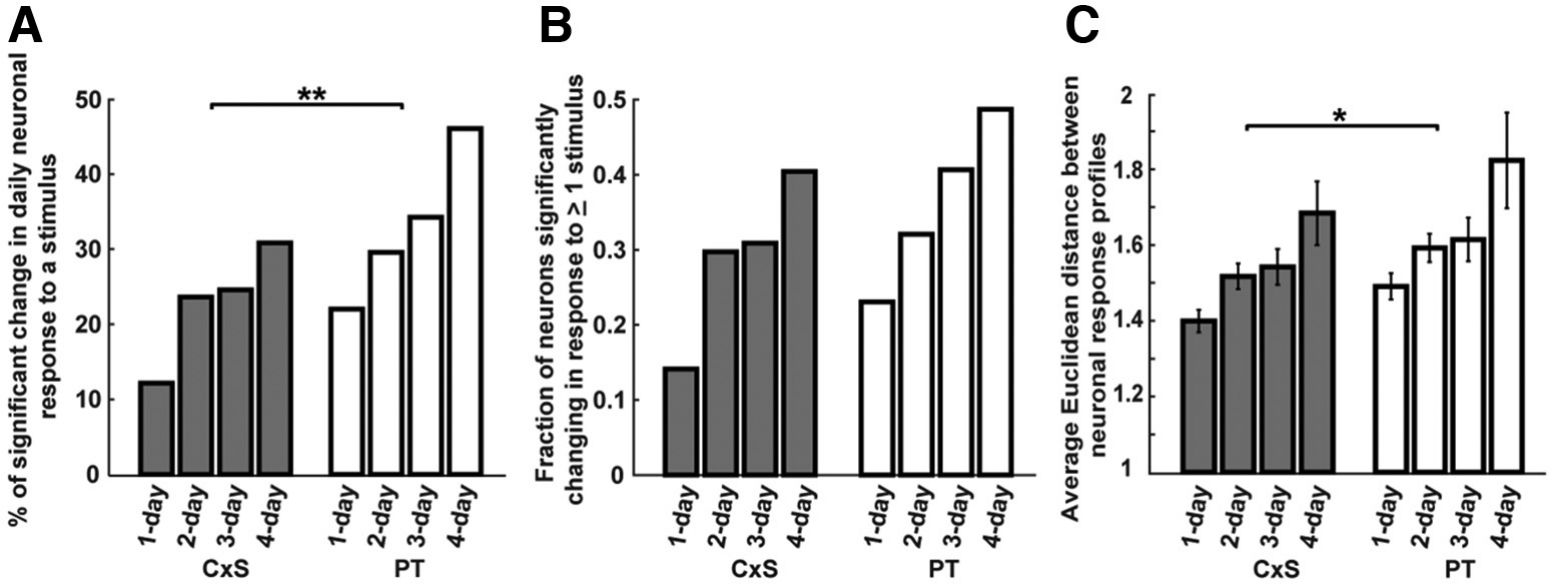
Auditory cortical responses to complex sounds are more stable than those to pure tones across multiple days. ***A***, Percentage of significant change in daily neuronal response to a given stimulus in the CxSs (gray bars) and PTs (white bars) protocol across varying daily intervals. **p = 0.0015 (bootstrap test; see Materials and Methods). ***B***, Fraction of neurons significantly changing in response to at least one stimulus in the protocol of CxSs (gray bars) and PTs (white bars) across varying daily intervals (*p* = 0.1084, bootstrap test; see Materials and Methods). ***C***, Average Euclidean distance between response profiles of a neuron for CxSs (gray bars) and PTs (white bars) across varying daily intervals. *p = 0.015 (two-way ANOVA).

10.1523/ENEURO.0031-22.2022.f1-4 Figure 1-4 Validation of neuron matching across days using image similarity analysis. ***A***, Cell bodies of neurons matched across a pair of consecutive days. Square dimensions = 39 × 39 pixels. ***B***, An example image similarity matrix corresponding to the cell bodies shown in ***A*** from a single focal plane, depicting the similarity for each neuron on day 1 compared against all neurons on day 2. Neurons manually matched have the same index assigned on each day. Color bar indicates the image similarity values. Following image registration, image similarity was calculated using the MATLAB structural similarity index (SSIM) for every pair of cell bodies across consecutive days (see Materials and Methods). ***C***, Distribution of normalized image similarity of manually matched neurons. The image similarity values for each neuron were divided by the maximum value across all its comparisons to yield the normalized image similarity value for each neuron. ***D***, Distribution of the percentage of neurons that showed the highest similarity rank to its manually matched neuron. Download Figure 1-4, TIF file.

Frequency bandwidth, spectral entropy, and spectrotemporal modulation were quantified for all sounds as attributes of sound complexity. Occupied frequency bandwidth quantifies the range of frequencies a sound is composed of and was calculated as the difference in frequency between the points where the integrated power crosses 0.5% and 99.5% of the total power in the spectrum. Spectral entropy of a sound quantifies how distributed its frequency content is and was calculated as the Shannon entropy of the normalized power distribution of the sound. Spectrogram autocorrelation of each sound measures the similarity of the frequency content of a sound across time bins and was calculated by temporally binning each spectrogram into 20 equally sized time bins (excluding brief periods of silence at the end of some sounds), resulting in column vectors that represent the power distribution of the sound at every time bin. We then calculated the Pearson correlations between all vectors and averaged the values of these correlations. Thus, the spectrogram autocorrelation of each sound inversely represents the degree of spectrotemporal modulation. The “spectrotemporal modulation index” was defined as one minus spectrogram autocorrelation.

### Data analysis

#### Preprocessing

Imaging data were run through the open-source Suite2p software package (Pachitariu et al., 2016) to correct for movement and neuropil signal, and to select neuronal regions of interest. To ensure reliable physiological measurements, we required that in any given imaging session, detected cell bodies show a compactness >0.8 and that the trace of their relative change in fluorescence (Δ*F*/*F*) shows a skewness >1.1 and clear transients (the experimenter was blind to sound responsiveness during the cell inclusion phase). A small minority of responses occurring during locomotion were excluded from all analyses (Extended Data Fig. 1-3). All further analysis was performed on the data preprocessed and output from Suite2p using custom-written MATLAB scripts (MathWorks, 2019a).

To identify the same neurons across imaging sessions, the average across-frames fluorescence image (with Suite2p median-filtering image enhancement) of each focal plane was used. The average fluorescence images of the same focal plane were then manually matched for the same neurons across days. We confirmed cell matching using fully automated image registration (MATLAB command: imregcorr) and calculation of structural similarity index (MATLAB command: ssim) of the cell bodies across days and found >95% agreement (Extended Data Fig. 1-4).

#### Two-photon imaging data analysis

The Δ*F*/*F* was defined for each neuron in a given imaging session as (*F*(*t*) – *F*_0_)/*F*_0_, where *F*(*t*) is the raw fluorescence signal of the cell at time *t*, and *F*_0_ is the median of the raw fluorescence signal across the session. The response magnitude of a given neuron to a sound was defined as the across-trials average Δ*F*/*F* within 0–1.5 s from sound onset. The responsiveness of a given neuron to each stimulus was determined using a bootstrap analysis. Specifically, the difference between the sound response magnitude across trials and the mean prestimulus (prestim) response magnitude [mean Δ*F*/*F* during in the prestim windows (−1.5 to 0 s) of all sounds in the protocol] was compared with a distribution of similar differences resulting from 1000 random shuffles of the sound responses and prestim responses. The neuron was considered responsive to a given stimulus if the difference between the real sound response and mean prestim magnitude was >97.5% of the shuffled differences and if the sound response magnitude was at least 10% greater than the prestim magnitude. On a given day, a neuron was considered sound responsive if it was responsive to at least one stimulus on that day (with Bonferroni’s correction for the number of stimuli).

To allow pooling changes in daily responses across neurons with different response magnitudes, the responses of each neuron to all stimuli across the 2 d of comparison were *z* scored before further analysis and statistical testing. For each comparison, a neuronal response to a given stimulus was included if the neuron was sound responsive on at least one of the days of comparison.

The significance of a change in response magnitude of a given neuron to a specific sound was quantified using a shuffle test. Specifically, the difference in mean response magnitudes between days was determined to be significant if the difference was >95% of the simulated differences generated from the random shuffling of trials across the days of comparison (*n*Shuffles = 1000) and in addition the magnitude of change was at least 10%. Using this method, we computed the significance of changes across four 1 d intervals (day 1 → day 2; day 2 → day 3; day 3 → day 4; day 4 → day 5), three 2 d intervals (day 1 → day 3; day 2 → 4; day 3 → day 5), two 3 d intervals (day 1 → 4; day 2 → day 5), and one 4 d interval (day 1 → day 5).

The percentage of significant change in daily neuronal responses to a stimulus was calculated as follows:

$$\frac{(\text{Number of sound responses that showed a significant change})\text{*}100}{\text{Total number of significant responses}}.$$

A neuron was determined to show significant change across days if it showed a significant change in response to at least one stimulus (after Bonferroni’s correction for the number of stimuli).

For a given neuron, we computed the average Euclidean distance between its response profiles (magnitude of responses across stimuli) across pairs of days using the following equation:

$$\sqrt{{\left(x_{1}-y_{1}\right)}^{2}+{\left(x_{2}-y_{2}\right)}^{2}+...+{\left(x_{n}-y_{n}\right)}^{2}},$$

where X_i_ equals the response of the neuron to stimulus *i* on the first day and *y_i_* equals the response of the neuron to stimulus *i* on the second day.

To test for the stimulus specificity of response change, we tested whether the day of recording (1 or 2) significantly interacted with the stimulus identity in determining response magnitude using a two-way ANOVA with interaction. The ANOVA output was used to compute the effect size (ω^2^) of the interaction term.

To test whether there is a significant difference between multiday or multistimuli proportions across CxSs and PTs (Fig. 1*F*; see also Fig. 4), we used a bootstrap analysis. Specifically, for each category across CxSs and PTs (see Fig. 4*A*, “1-day”), we derived a distribution of 10,000 randomly simulated PT proportions given the probability of the corresponding CxS category. The *p*-value was calculated as the fraction of “CxS-simulated” PT probabilities that were equal to or higher than the real PT probabilities across categories.

#### Statistical tests

We used statistical tests at a *p* < 0.05 significance level and α = 0.05 for all comparisons unless otherwise indicated (Table 1).

## Results

To quantify the degree of stability of auditory cortical representations of PTs and CxSs, we conducted two-photon calcium imaging of identified excitatory neuronal ensembles in L2/3 of the AC (Extended Data Fig. 1-1) in 10 awake head-fixed Thy1-GCaMP6f mice across days. As the degree of sound novelty influences response magnitude in AC (Ulanovsky et al., 2003, 2004; Nelken, 2014; Kato et al., 2015; Parras et al., 2017; Heilbron and Chait, 2018), we familiarized the mice to the experimental sound protocols for 3 consecutive days while being head fixed under the two-photon microscope before data acquisition commenced (Fig. 1*A*). During this habituation period, in each animal, three optical focal planes were chosen and registered with respect to the overlying blood vessel pattern to allow for repeated imaging of the same neurons across days (Fig. 1*B*).

From day 1 to day 5 of the experiment, we imaged the daily responses of identified neuronal ensembles to eight PTs of varying frequencies and eight CxSs. The CxSs consisted of animal vocalizations and environmental sounds that broadly overlapped in frequency content with the PTs, while having significantly higher frequency bandwidth, spectral entropy, and spectrotemporal modulation (Fig. 1*C*,*D*; see Materials and Methods). As expected, AC neurons responded to both PTs and CxSs with sound-triggered transients in Δ*F*/*F* (Fig. 1*E*). We first compared the degree of overall sound-evoked responsiveness to CxSs and PTs across the population. We found that response magnitudes to PTs and CxSs were not significantly different (Fig. 1*F*) and that the rate of responsive neurons to PTs and CxSs were also not significantly different (Fig. 1*G*). Thus, our chosen set of PTs and CxSs evoked similar magnitudes and rates of responses among L2/3 AC excitatory neurons. Responsiveness, response magnitude, and trial-to-trial consistency were not underestimated by our imaging sample rate (Extended Data Fig. 1-2).

We next quantified the degree of stability of these neuronal responses by comparing the responses of identified neurons across pairs of consecutive days. The identical variation in daily experimental and physiological conditions for PTs and CxSs allowed us to compare the relative degrees of change in responses between the two sound protocols. We observed that while most responses of individual AC neurons showed stability across days, some displayed significant daily variation (Fig. 2*A*). To measure changes in sound responses, we first focused on the responses of individual neurons to individual stimuli across pairs of consecutive days and restricted our analyses to responses that were significant in at least one of the two days. Across this population, we found that while the majority of responses were stable across days, 22.01% (114 of 518) of significant responses to PTs showed a significant change in response magnitude across successive days (Fig. 2*B*). These results suggest that, underlying a generally stable representation, responses of AC neurons to PTs show a moderate degree of daily dynamics. Interestingly, however, only 12.15% (66 of 543) of significant responses to CxSs showed a significant change in magnitude across the same time interval (Fig. 2*B*). This proportion of daily response change to CxSs was significantly lower than that of PTs (Fig. 2*B*), suggesting that AC responses to CxSs are more stable than responses to PTs across days. The degree of stability of CxSs with well-defined spectral centroids at <10 kHz (Cricket, Chiffchaff, and Macaque) did not significantly differ from those of more distributed spectra (Glass, Shrew, Thump, Scratch, and Water; 12.36% vs 12.05%, respectively; *p* = 0.92, χ^2^ test for proportions).

10.1523/ENEURO.0031-22.2022.f2-1 Figure 2-1 Relationship between the changes of a neuron in responsiveness to CxSs and PTs. ***A***, Correlation of the average Euclidean distance of the response profile of a neuron from one day to the next for CxSs and PTs (*r* = –0.037, *p* = 0.74, Pearson’s correlation test). ***B***, Correlation of the change in response magnitude for each responsive neuron to CxSs with changes in response magnitude to PTs that had overlapping frequencies with the CxSs (left; *r* = 0.168, *p* = 10^−6^, Pearson’s correlation test) and with PT stimuli that had minimal overlapping frequencies with CxSs (right; *r* = 0.149, *p* = 10^−5^, Pearson’s correlation test). The correlations did not significantly differ (*p* = 0.341, Fisher’s *z* test). PTs with frequency overlap with the CxSs were determined as the three to four PT frequencies that maximally overlapped with the power spectrum of the CxSs. Download Figure 2-1, TIF file.

As a complementary approach, we quantified a similar measure at the single-neuron level rather than the single-stimulus level. To this end, we calculated the fraction of sound-responsive neurons that exhibited a significant change in response magnitude to at least one of the eight PTs or CxSs for each pair of consecutive days. Consistent with our findings at the single-stimulus level, we found that the fraction of neurons showing a significant change in response to CxSs was significantly lower than that to PTs (Fig. 2*C*).

To quantify the stability/plasticity of sound responses at the level of response profiles across stimuli, we computed for each neuron the Euclidean distance between its response profile (to either PTs or CxSs) on one day and that of the next day. A larger Euclidean distance reflected a higher degree of response change across stimuli. Consistent with the findings above, we found that the Euclidean distance between daily response profiles to PTs was significantly higher than those to CxSs (Fig. 2*D*). There was no significant correlation between the Euclidean distance of the same neurons to PTs and CxSs (Extended Data Fig. 2-1*A*) and changes in responses to CxSs were not significantly more strongly correlated with changes in frequency-overlapping PTs compared with frequency-nonoverlapping PTs (Extended Data Fig. 2-1*B*). Together, these findings across varying quantification methods indicate that AC neuronal responses to CxSs are more stable than those to PTs across consecutive days.

A change in the response profile of a neuron across days may include a change in response gain, manifesting as similar changes in response magnitude across stimuli, or it may be stimulus-specific, reflecting a change in the neuronal sound selectivity (Fig. 3*A*). To test whether changes in responses to PTs and CxSs differed in the nature of change, we compared the degree of stimulus specificity of response change for each of the stimuli classes. We tested for the responses of each neuron, whether there was a significant interaction between the day of recording and the different stimuli. A significant day–stimulus interaction indicates that responses to the different stimuli were differentially modulated across days, reflecting stimulus specificity in response change. We found that a significantly higher proportion of neurons showed stimulus specificity in daily changes in responsiveness to PTs compared with CxSs (Fig. 3*B*). Further, the strength of the day–stimulus interaction was significantly higher for PTs than for CxSs (Fig. 3*C*). These findings indicate that in addition to showing higher overall rates of daily change in responsiveness, the changes in responses to PTs were more stimulus-specific, and therefore reflected a higher degree of change in sound selectivity, compared with CxSs.

Finally, we investigated how the rates of change across pairs of days relate to rates of change across longer durations. To this end, we quantified the changes in responsiveness in a similar manner across intervals of 1–4 d. We found that the degree of response plasticity increased with increasing time interval between days for both CxSs and PTs (Fig. 4*A*,*B*). Moreover, the elevated rates of change in responses to PTs compared with CxSs that were observed across pairs of days also manifested across these intervals (Fig. 4*A*). The fraction of neurons showing a significant change to at least one stimulus showed a similar trend, though it did not reach significance (Fig. 4*B*). Last, the Euclidean distance between the PT response profiles was significantly higher than that of CxSs across these intervals (Fig. 4*C*). Consistent with our previous results, this suggests that AC representations of CxSs are more stable compared with PTs over a range of daily time intervals.

## Discussion

In this study, we used two-photon calcium imaging to record the degree of stability and plasticity of sound-evoked responses of L2/3 AC excitatory neurons to PTs and CxSs across days. We found that most responses to both PTs and CxSs were stable, with a moderate but significant degree of change across pairs of consecutive days. Importantly, we report that responses to CxSs exhibited significantly enhanced stability across days compared with PTs. Furthermore, the structure of response profiles to PTs exhibited larger degrees of change than to CxSs across days, as evidenced by a higher degree of stimulus-specific changes. Finally, we found that the enhanced degree of stability in CxS representations generalizes to longer daily time intervals.

Our findings of a significant degree of ongoing daily changes in auditory cortical representations of both CxSs and PTs add to a number of recent studies describing “representational drift” in other sensory modalities (Peron et al., 2015; Ranson, 2017; Rule et al., 2019; Deitch et al., 2021; Pérez-Ortega et al., 2021; Schoonover et al., 2021). Together, these studies point to a potential common principle, by which, despite the well established link between perception and cortical function (Bergman, 1990; Chait et al., 2010; Chapuis and Wilson, 2011; Leopold, 2012; Frégnac and Bathellier, 2015; Kuchibhotla and Bathellier, 2018; Ceballo et al., 2019; Lee and Rothschild, 2021), a stable sensory perception does not rely on fixed cortical sensory representations. Instead, representational dynamics may reflect a general principle of cortical function. Indeed, the locally heterogeneous organization of AC L2/3 ensembles has been suggested to be well suited to support rapid synaptic reorganization in response to changing environmental conditions (Bandyopadhyay et al., 2010; Rothschild et al., 2010, 2013; Bathellier et al., 2012; Kanold et al., 2014; Kato et al., 2015; Rothschild and Mizrahi, 2015; Maor et al., 2016; Francis et al., 2018; Liu et al., 2019; Liu and Kanold, 2021). The question of whether sound representations in the thalamorecipient L4 are more stable than those in L2/3 remains for future studies.

While auditory cortical representations of both classes of sounds exhibited significant degrees of daily change, representations of CxSs were significantly more stable compared with PTs across quantification methods. These findings likely result from the differences in the acoustic properties of these stimuli. In particular, CxSs are decomposed into narrow frequency channels at the cochlea, and reconstructing their wideband frequency contents throughout the auditory pathway requires reintegration across frequency channels. In contrast, a pure tone evokes responses in a narrower channel throughout the auditory system. If daily variation in responses is at least partly independent in different frequency channels, integration across frequency bands as needed to represent CxSs may “average out” some of this variation compared with that of PTs. Thus, spectrotemporal integration may give rise to enhanced longitudinal stability of CxSs in the AC. Future studies could directly test this possibility by, for example, measuring the stability of representations of noise with systematically varying bandwidths. An alternative acoustic property that may determine the degree of AC stability is based on temporal rather than spectral integration. In particular, temporal modulations in the complex sounds may “reset” neuronal responses multiple times within a stimulus, such that the enhanced degree of overall stability is because of temporal averaging of per modulation fluctuations. This possibility could be tested using sequences of amplitude-modulated tones, which have temporal modulation without spectral bandwidth.

Beyond the higher degrees of change in responses to PTs compared with CxSs, we also found that PT response changes were more stimulus-specific than those of CxSs. These findings suggest that changes in responses to CxSs tended to be shaped more by global gain factors while changes in responses to PTs tended to reflect stimulus-tuning changes to a larger degree. If changes to CxSs are correlated with changes to the tones that make up the CxSs, this finding may be influenced by the frequency overlap between CxSs, which is not the case for PTs. Although our finding that responses to CxSs do not significantly change as to their frequency-overlapping tones (Extended Data Fig. 2-1) argues against this possibility, the experiments described above could directly test it.

Beyond the acoustic differences between CxSs and PTs, a combination of evolution and previous experience may also have contributed to the enhanced stability of AC representations of CxSs compared with PTs. Future studies may test this hypothesis by comparing the degree of AC stability to sounds with similar spectrotemporal complexity but varying ethological relevance.

Our findings raise the question of whether enhanced stability of AC representations of CxSs are linked with the enhanced perceptual stability of these sounds. As the AC is important for sound perception in both humans (Kaga et al., 1997; Griffiths, 2012) and animal models (Ohl et al., 1999; Harrington et al., 2001; Rybalko et al., 2006; Frégnac and Bathellier, 2015; Kuchibhotla and Bathellier, 2018; Ceballo et al., 2019), it is tempting to speculate based on our findings that behavioral measures of perceptual stability, such as sound recognition across days, would be higher for CxSs compared with PTs. Testing this speculation may have important implications as PTs are not just widely used in auditory research but are also the standard in studies using classical conditioning and other learning paradigms.

## Synthesis

Reviewing Editor: Christine Portfors, Washington State University Decisions are customarily a result of the Reviewing Editor and the peer reviewers coming together and discussing their recommendations until a consensus is reached. When revisions are invited, a fact-based synthesis statement explaining their decision and outlining what is needed to prepare a revision will be listed below. The following reviewer(s) agreed to reveal their identity: Daniel Llano.

The reviewers agree that this is an interesting manuscript addressing an important question of sound processing in the auditory cortex. There are however some major issues that need to be addressed. It is critical that the manuscript convincingly demonstrate that the neurons imaged across multiple days are in fact the same neurons. It is also critical that the frame rate used for imaging be confirmed. If it really was 3 fps instead of the commonly used 30 fps, this is a major problem for sampling temporally patterned responses and likely impacted the results. Finally, the authors should provide evidence that their images come from primary auditory cortex.

Detailed comments:

1.The authors used galvo scanning at 3 frames/second and the GCaMP6f fluorescence indicator. This yields only 1 data point every 333 ms, while GCaMP6f has a fast response time (< 500 ms per 10 action potentials). Furthermore, the sounds in this study were < 500 ms. Thus, the authors might have entirely missed responses to some of the sounds, or underestimated response magnitudes. This is particularly problematic in a study on the stability of neural responses.

2.This study relies on the reader believing that the same neurons were in fact recorded from across 5 days, yet the authors describe the process of selecting the same neurons as largely “manual”. The similarity of responses in the example neurons builds confidence, however, the authors need to quantify their certainty in the proportion of neurons that were tracked across days. If 1/3 of the neurons changed every day, that could easily introduce a bias in metrics of stability. Especially when comparing responses to pure-tones vs complex sounds, where the responsiveness to pure tones depends on the tuning of neurons in a field of view.

3.It is clear that the authors recorded from auditory cortex, yet considering the growing literature indicating very different roles of primary vs higher-order auditory cortex in processing complex sounds and acoustic features (eg. work from the Kato, Kanold, and Barkat labs), it is critically important for this study to clarify where the recordings were made in auditory cortex.

4.Considering that acoustic complexity is central to the manuscript, the extended data should be moved to figure 1. In addition, the authors should explain how each metric indicates “complexity”.

Minor Comments

1.In general, the writing could do with less speculation and less vague word choices.

1a.Lines 38-39: “unique role” is too vague of a characterization

1b.Lines 43-43: “Physiological support for a unique representation of CxS” - not clear what is meant by a “unique representation"

1c.Lines 51-52: It is an overstatement to say that, “the role of the AC in representing CxS is well established"

1d.Lines 54-56: what is “their” referring to?

1e.Lines 63-64: Too strongly worded

1f.Lines 314-316: too much speculation

1g.Lines 336-344: too much speculation

2.Line 142: what is an “enhanced average”?

3.The authors mention rejecting data during locomotion-how much data was rejected and how did they account for the resulting different number of kept trials per sound?

4.How much did the results depend on the criterion for 10% greater activity than in pre-stim silence? Why was this criterion for “responsiveness” used in addition to the statistical tests?

5.Fig 2 panel d: please add “between neuronal response profiles” to the axis title

6.Considering that some of the complex sounds have most energy <10 Khz, it would be edifying to compare the stability of complex sounds with well defined spectral centroids vs more distributed spectra.

7. It may be that increased stability to CxS sounds simply represents the fact these responses are dependent upon a broader array of inputs being activated. Therefore, the increase in the stability simply represents a greater robustness to dropout of components of the signal due to plasticity over time. This could be tested by incorporating a white noise stimulus, which would not be behaviorally relevant, but activates many inputs to these neurons. Alternatively, the increased stability to CxS could be due to temporal modulations that “reset” the neurons multiple times within a stimulus. This could be tested using AM tones. I am not suggesting that the authors need to do these experiments, but should discuss these possibilities.

8. Was there any relationship between the change in a neurons responsiveness to PTs and its responsiveness to CxS? This could be examined by examining correlations between changes to PTs and changes to CxS per neuron. This would give you some idea if changes to CxS response properties were due to dropout or enhancement of responses to particular tones.

9. The argument for changes in response selectivity in PTs vs. CxS in Fig 3 is dependent upon the relative similarity of PT stimuli vs. CxS stimuli. PT stimuli have zero spectral overlap while CxS stimuli have broad spectral overlap. Therefore, it is more likely, based on heterogeneity of stimuli, that you would see heterogeneous response changes to PT than CxS stimuli. This should be discussed.

10. Please clarify what is happening during the 3-day habituation period. Are the animals being exposed to the same PT and CxS stimuli as during days 1-5? If so, how many repetitions each?

11. Please specify the approximate time-of-day of experiment and if animals were clock (day/night) reversed (“standard” is stated - please clarify).

12. Title: “Enhanced” is vague and needs a clarifying phrase. I would suggest “Enhanced stability of complex sound representations relative to simple sounds in the auditory cortex"
