# Supplementary figures and images for: Enhanced Stability of Complex Sound Representations Relative to Simple Sounds in the Auditory Cortex
Source: eNeuro. 2022 Aug 1;9(4):ENEURO.0031-22.2022. doi: 10.1523/ENEURO.0031-22.2022 (PMC9347310; doi:10.1523/ENEURO.0031-22.2022)

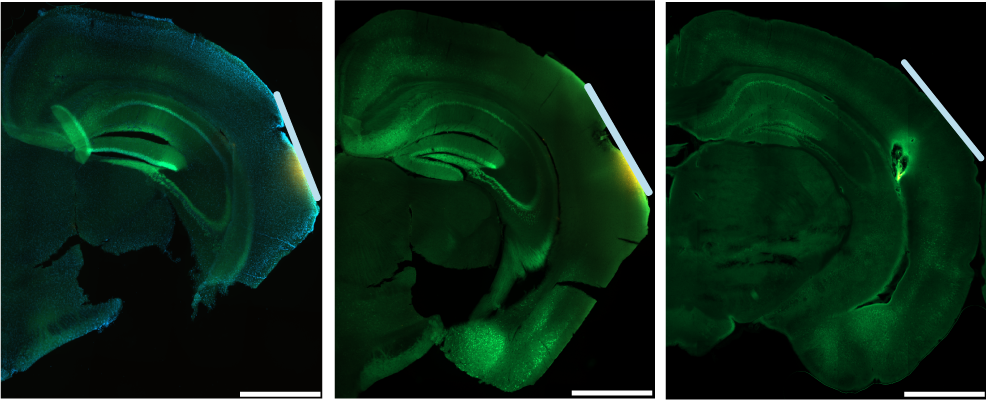

Supplement: Figure 1-1 — Histological verification of imaging location. Representative images of coronal brain sections from three animals used in this study. Following the completion of the experiments, mice were killed with an overdose of xylazine (10 mg/kg, i.p.) and the imaging cranial window was removed. Using a nanoFil needle (Hamilton), we injected 1 μl of Dil Tracer (catalog #D282, Thermo Fisher Scientific) into the site of imaging identified by blood vessel patterns and covered the brain surface for 5 min to maximize labeling and prevent fluorescence loss caused by the perfusion during tissue fixation with 4% PFA. The extracted brains were kept in PFA for 3 d and then transferred to 30% sucrose solution for another 3–4 d before cryosectioning. The brains were sliced in 50-μm-thick sections and preserved with Fluoroshield mounting medium with DAPI (Abcam). Recording site confirmation was done by imaging tissue sections positive for GCaMP, DAPI, and Dil fluorescence. Dil fluorescence trace from brain sections were cross-referenced with the Allen Mouse Common Coordinate Framework using NeuroInfo software (MBF Bioscience). The location of the cranial window is indicated on each brain slice, and the specific site of imaging is marked by DiI in yellow. Scale bar, 1000 μm. Download Figure 1-1, TIF file. [file enu-eN-NWR-0031-22-s02.tif]

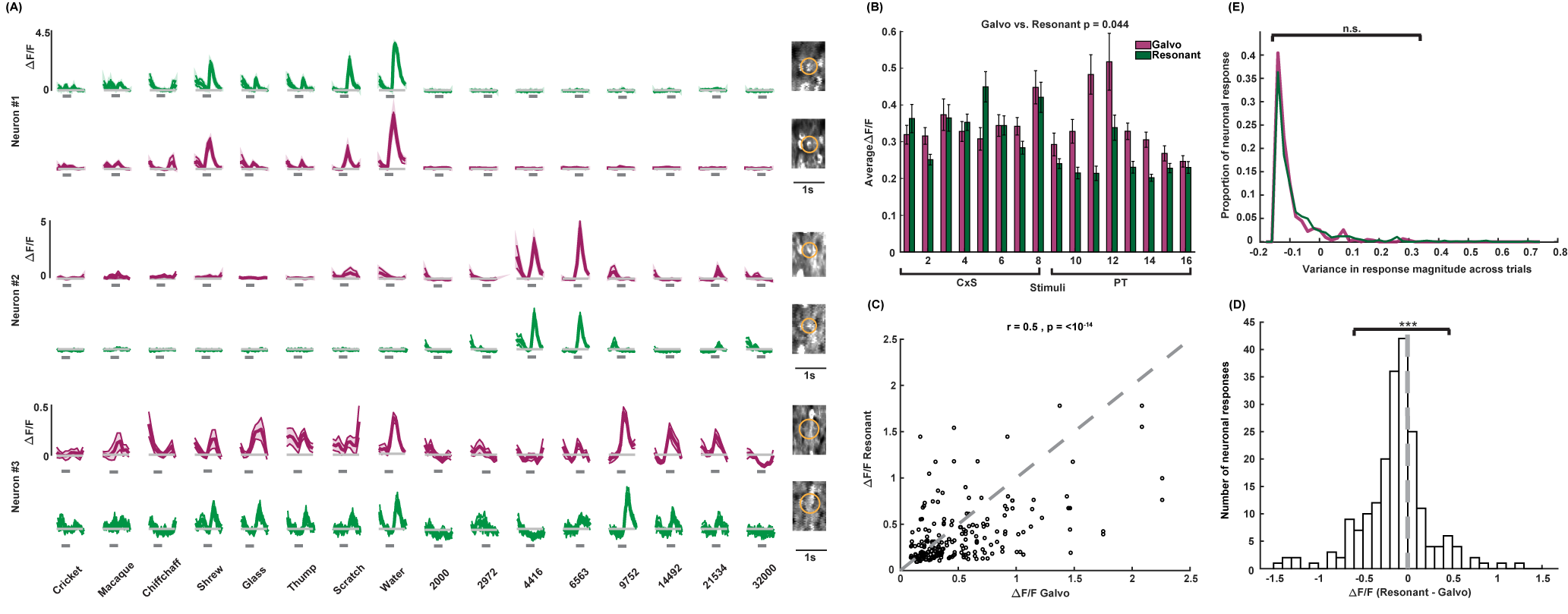

Supplement: Figure 1-2 — Comparison of auditory cortical responses to CxS and PT in galvo and resonant scanning modes. A, Responses of three representative neurons in galvo (in maroon) and in resonant (in green) scanning modes to CxS and PT stimuli (corresponding stimuli are indicated at the bottom of the panel). Shaded area marks the mean ± SEM across trials. The gray bar below each response indicates the stimulus time (0.5 s). Calibration: 1 s. The cell body of the neuron as imaged in the two scanning modes is highlighted on the right. B, Average sound response magnitude (mean ΔF/F across all trials over the stimulus window) across both imaging modes in response to complex sounds (Stimuli #1–8) and pure tones (Stimuli #9–16). Error bars indicate the mean ± SEM. Each focal plane was imaged in galvo and resonant scanning modes alternating twice, and all pairs of consecutive imaging sessions were included in the comparison between galvo and resonant scanning modes. The number of neurons across all pairs of scanning modes (with repetitions): galvo, 576; resonant, 540; p = 0.044 (two-way ANOVA). In a separate analysis, we found that the likelihood of neurons to be responsive to a specific CxS and a specific PT using resonant scanning was 7.74% (41 of 530) and 7.24% (38 of 525), respectively. Using galvo scanning, these values were slightly higher, at 9.23% (53 of 574) and 10.44% (55 of 527), respectively, suggesting that significant responses were not underestimated by the use of galvo scanning. C, Correlation of the response magnitudes of individual responsive neurons (mean ΔF/F across all trials over the stimulus window) to individual stimuli (both CxS and PT included) in galvo and resonant scanning modes. Neurons were matched across scanning modes using an automated MATLAB algorithm (https://github.com/ransona/ROIMatchPub) and then validated by visual inspection. Dashed gray line represents the diagonal. Correlation coefficient = 0.5, p = 2.36 × 10^−14^ (Pearson’s correlation). D, Distributio [file enu-eN-NWR-0031-22-s03.tif]

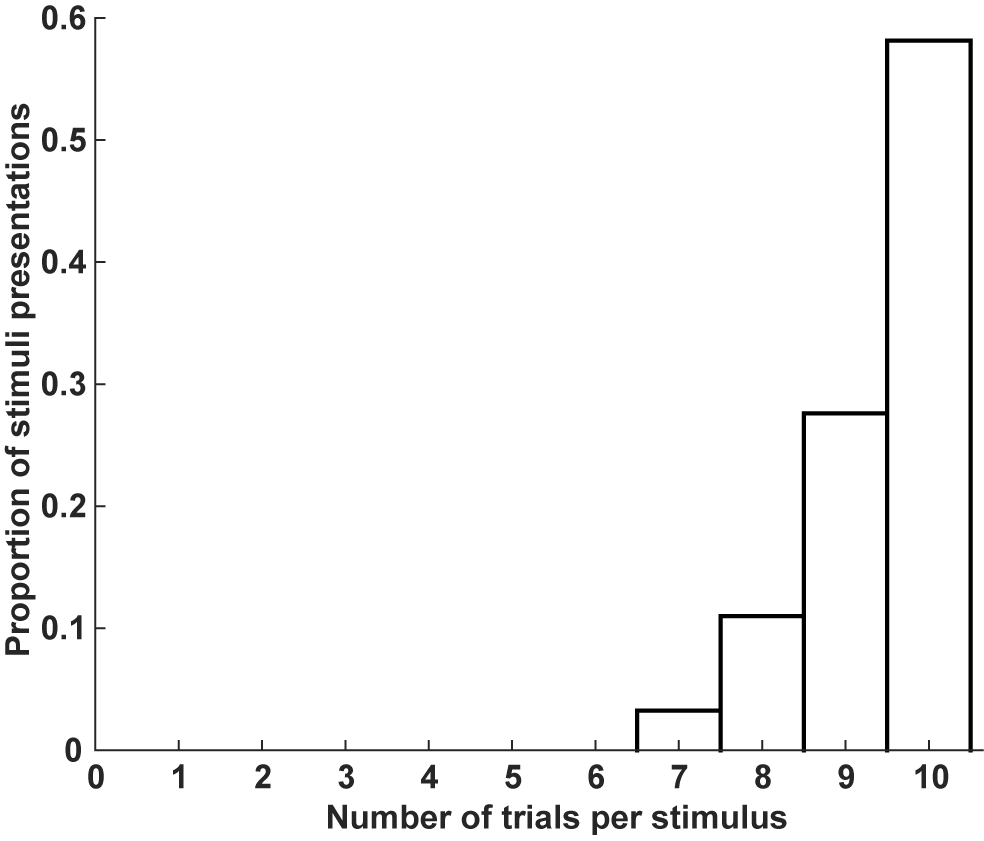

Supplement: Figure 1-3 — Distribution of number of trials included per stimulus across the dataset following trial exclusion due to locomotion. Download Figure 1-3, TIF file. [file enu-eN-NWR-0031-22-s04.tif]

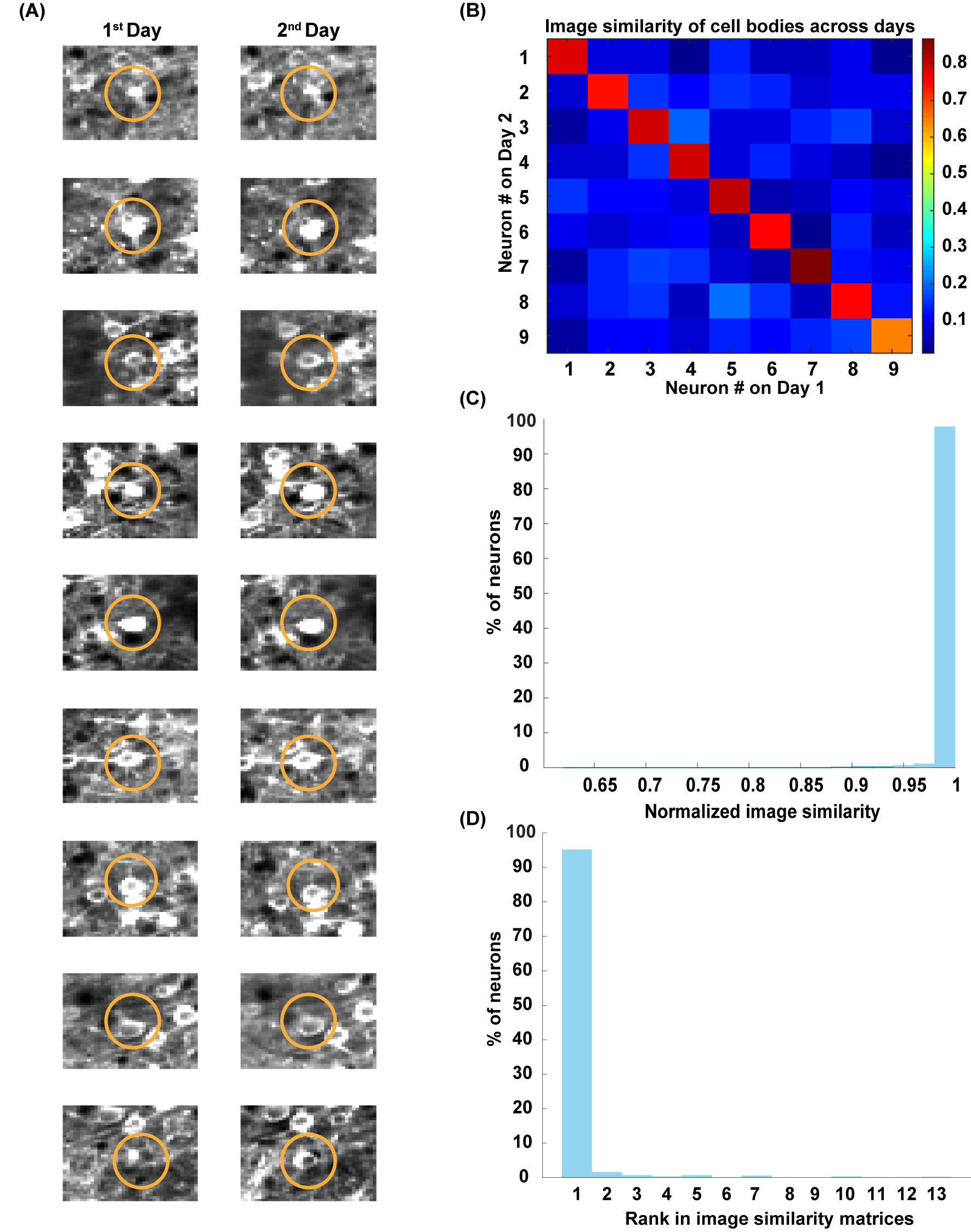

Supplement: Figure 1-4 — Validation of neuron matching across days using image similarity analysis. A, Cell bodies of neurons matched across a pair of consecutive days. Square dimensions = 39 × 39 pixels. B, An example image similarity matrix corresponding to the cell bodies shown in A from a single focal plane, depicting the similarity for each neuron on day 1 compared against all neurons on day 2. Neurons manually matched have the same index assigned on each day. Color bar indicates the image similarity values. Following image registration, image similarity was calculated using the MATLAB structural similarity index (SSIM) for every pair of cell bodies across consecutive days (see Materials and Methods). C, Distribution of normalized image similarity of manually matched neurons. The image similarity values for each neuron were divided by the maximum value across all its comparisons to yield the normalized image similarity value for each neuron. D, Distribution of the percentage of neurons that showed the highest similarity rank to its manually matched neuron. Download Figure 1-4, TIF file. [file enu-eN-NWR-0031-22-s05.tif]

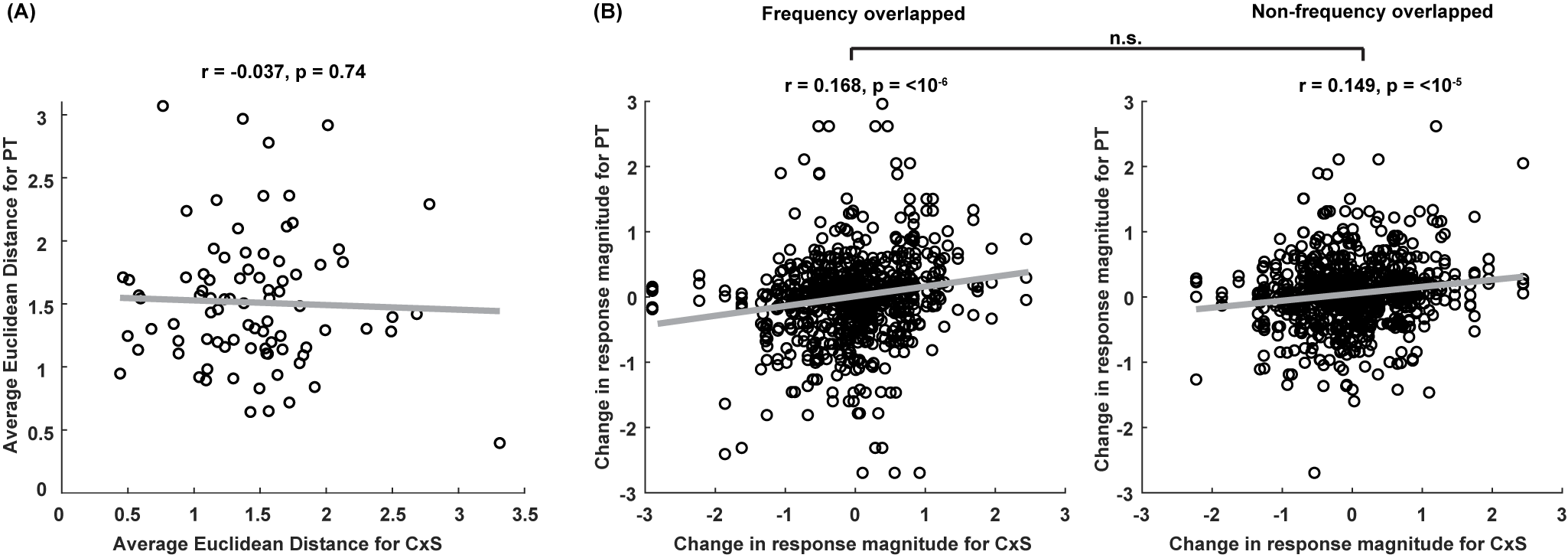

Supplement: Figure 2-1 — Relationship between the changes of a neuron in responsiveness to CxSs and PTs. A, Correlation of the average Euclidean distance of the response profile of a neuron from one day to the next for CxSs and PTs (r = –0.037, p = 0.74, Pearson’s correlation test). B, Correlation of the change in response magnitude for each responsive neuron to CxSs with changes in response magnitude to PTs that had overlapping frequencies with the CxSs (left; r = 0.168, p = 10−6, Pearson’s correlation test) and with PT stimuli that had minimal overlapping frequencies with CxSs (right; r = 0.149, p = 10−5, Pearson’s correlation test). The correlations did not significantly differ (p = 0.341, Fisher’s z test). PTs with frequency overlap with the CxSs were determined as the three to four PT frequencies that maximally overlapped with the power spectrum of the CxSs. Download Figure 2-1, TIF file. [file enu-eN-NWR-0031-22-s06.tif]
